# EUPATI and Patients in Medicines Research and Development: Guidance for Patient Involvement in Ethical Review of Clinical Trials

**DOI:** 10.3389/fmed.2018.00251

**Published:** 2018-09-07

**Authors:** Ingrid Klingmann, Andrea Heckenberg, Kay Warner, David Haerry, Amy Hunter, Matthew May, Wolf See

**Affiliations:** ^1^EUPATI, European Forum for Good Clinical Practice, Brussels, Belgium; ^2^EUPATI, Ethics Committee, Medical University Vienna, Vienna, Austria; ^3^EUPATI, GlaxoSmithKline, Brentford, United Kingdom; ^4^EUPATI, European Aids Treatment Group, Brussels, Belgium; ^5^EUPATI, Genetic Alliance UK, London, United Kingdom; ^6^EUPATI, European Patients Forum, Luxembourg, Luxembourg; ^7^EUPATI, BAYER, Berlin, Germany

**Keywords:** patient engagement, patient involvement, guideline, ethical review, clinical trial, medicines development, EUPATI

## Abstract

Involvement of patients in the research and development process (R&D) of new medicines—in all areas of indications—today is a widely accepted strategy in pharmaceutical industry to ensure relevance and suitability of the treatment under development. This may consist in, but is not limited to, patient input to achieve more patient-friendly protocol design, endpoint, and comparator selection as well as disease-adapted study conditions in a pre- or post-marketing clinical trial. Ethical aspects and especially the balance of benefit and risk in a clinical trial are frequently judged differently by clinical researchers, regulators, ethics committees, and patients due to their different focus. The final assessment of the ethical aspects of a planned clinical trial is provided by an independent ethics committee consisting of physicians and other experts in healthcare and clinical trial methodology as well as of lay persons. The participation of patients in ethics committees is a much-discussed concept, its suitability disputed in many countries, and only limited experience on best practices is available. In order to be effective and yield the best results for all stakeholders, integration of patients into the medicines development process needs to be structured and governed by clear, mutually agreed rules and modes of operation. Communication and collaboration processes need to be systematically implemented to establish transparency, trust and respect between those developing new medicines and their users, respectively between those involved in design and approval of clinical trials and participants. In particular agreement on the ethical aspects of a clinical trial and/or its overall ethical acceptability is a prerequisite before the start of a clinical trial. Existing codes of practice for patient involvement with various stakeholders do not comprehensively cover the full scope of R&D, with the exception of more general statements on interaction. Overarching guidance on meaningful and ethical interaction is missing. One specific aim of the European Patients' Academy on Therapeutic Innovation (EUPATI) was to close this gap through the development of guidance documents for ethics committees, pharmaceutical industry-led medicines R&D, regulatory authorities, and health technology assessment (HTA). This EUPATI “Guidance for patient involvement in ethical review of clinical trials” gives practical recommendations for ground rules and lists options for conditions and practices for involving patients in the work of ethics committees to enable trustful and constructive collaboration whatever the national (legal) framework for patient involvement in ethics committees might be. The guidance sets the collaboration of patients in ethics committees in the broader context of relevance and opportunities for patient input on ethics in the overall medicines R&D and specifically the overall clinical trial process from concept development to trial result reporting in lay summaries. In addition to a presentation of the full text of the Guidance, this article aims at providing additional background information on the development process of the Guidance, as well as insight into the current debate on this topic.

## Introduction

In line with the Declaration of Helsinki (1), the ICH-GCP guideline (2), and the European Clinical Trial Directive 2001/20/EC (3) all European member states and in fact all other countries involved in clinical trials have established an ethics committee system that allows thorough ethical review of biomedical research in human subjects. Minimal standards for composition, operations, and procedures of ethics committees have been defined in the ICH-GCP guideline. However, countries have chosen to implement varied ethical review systems, performed by ethics committees with very different composition and focus of their work. Nevertheless, in all countries, their aim is to provide sponsors and researchers with a thorough, unbiased ethical opinion formed by a multi-disciplinary committee of scientific experts in their field, supported by lay person input. In a few countries also patients are members of an ethics committee, either based on national legal requirements or—more recently—on the initiative of the individual ethics committee. Patient input sought ranges from “naïve” views on legibility and understandability of the patient information sheet and informed consent form in some countries to ethical expertise concerning acceptability of protocol design, study conditions and benefit-risk balance by patients well versed in the medicines development process. Experience with the benefit of this patient involvement is mixed, ranges from “not really helpful” to “lip service” to “very helpful” to “essential,” based on information collected in surveys, workshops and webinars organized by EUPATI, the European Patients' Academy on Therapeutic Innovation^1^. Ethics committees not involving patient members argue that, in contrast to all other members, patient members would not be “neutral” but “biased” as they would be “concerned.” Also the practical effort of finding the right patients—either those with the respective disease or knowledgeable patients who can provide the patients' views irrespective of the indication—would be difficult to manage.

As a result of the growing interest and willingness of patients to contribute to a more patient-centered medicines development process more and more patients are prepared to join ethics committees. They want to provide their expertise of living with the disease, their insight into potential benefits and risks of the new medicine and feel comfortable contributing additional aspects. However, many fundamental concerns and open questions on the role and expected contributions of patients in ethics committees, on their required level of expertise in the medicines development process, their representativeness for all patients with this disease, and on optimal organizational and financial conditions for this collaboration exist on ethics committees' as well as on patients' sides as no agreed regulatory or legal framework has been worked out for such collaboration on an international basis.

However, involvement of patients in the work of ethics committees is not their only possibility to improve the ethical acceptability of clinical trials for participating patients. The experience and opinion gathering activities performed in EUPATI revealed that patients can and should contribute important insights to a continuum of activities in the medicines R&D process. Specific patient input on ethical aspects should be enabled all along the clinical trial preparation process because decisions pertaining to ethics are made at many timepoints before the submission of the clinical trial dossier to the ethics committee.

The Guidance presented here has been developed as one of four EUPATI guidances addressing key areas of interaction and generally agreed collaboration principles for different stakeholders and patients in the medicines development process. An introductory part provides information on the background of EUPATI and the benefits of patient education and engagement throughout the medicines development process. Besides definitions of joint values, this Guidance provides suggestions for aspects in R&D, specifically the different phases of the clinical trial process that benefit from patient contributions, as well as the required level of patient expertise in their area of indication and medicines development methodology. In the main part of the guidance an overview of the options for involvement of patients in ethics committees is presented that reflects the diverse and rapidly changing situation in Europe. Concrete proposals for ethically correct, trustworthy and transparent patient involvement in ethics committees are given, based on the multi-stakeholder work of the EUPATI guidance task force, feedback from workshops, webinars, and a survey on experiences and expectations on patient involvement in the ethical review of clinical research projects as well as a broad public consultation.

Although this Guidance can be found on the EUPATI website this article with the embedded Guidance text represents the formal publication of this relevant EUPATI project result.

## The guidance text

### The EUPATI guidance on patient involvement in ethical review of clinical trials

Background information identical in all four EUPATI Guidances:

#### Overarching principles for patient involvement throughout the medicines research and development process

The European Patients' Academy (EUPATI) is a pan-European Innovative Medicines Initiative (IMI) project of 33 organizations with partners from patient organizations, universities, not-for-profit organizations, and pharmaceutical companies. Throughout EUPATI the term “patient” references all age groups across conditions. EUPATI does not focus on disease-specific issues or therapies, but on the process of medicines development in general. Indication-specific information, age-specific or specific medicine interventions are beyond the scope of EUPATI and are the remit of health professionals as well as patient organizations. To find out more visit www.eupati.eu/.

The great majority of experts involved in the development and evaluation of medicines are scientists working both in the private and public sector. There is an increasing need to draw on patient knowledge and experience in order to understand what it is like to live with a specific condition, how care is administered and the day-to-day use of medicines. This input helps to improve discovery, development, and evaluation of new effective medicines.

Structured interaction between patients of all age groups and across conditions, their representatives and other stakeholders is necessary and allows the exchange of information and constructive dialogue at national and European level where the views from users of medicines can and should be considered. It is important to take into account that healthcare systems as well as practices and legislation might differ.

We recommend close cooperation and partnership between the various stakeholders including healthcare professionals' organizations, contract research organizations, patients', and consumers' organizations^2^, academia, scientific, and academic societies, regulatory authorities and health technology assessment (HTA) bodies and the pharmaceutical industry. Experience to date demonstrates that the involvement of patients has resulted in increased transparency, trust and mutual respect between them and other stakeholders. It is acknowledged that the patients' contribution to the discovery, development and evaluation of medicines enriches the quality of the evidence and opinion available (4).

Existing codes of practice for patient involvement with various stakeholders do not comprehensively cover the full scope of research and development (R&D). The EUPATI guidance documents aim to support the integration of patient involvement across the entire process of medicines research and development. These guidance documents are not intended to be prescriptive and will not give detailed step-by-step advice.

EUPATI has developed these guidance documents for all stakeholders aiming to interact with patients on medicines research and development (R&D). Users may deviate from this guidance according to specific circumstances, national legislation, or the unique needs of each interaction. This guidance should be adapted for individual requirements using best professional judgment.

There are four separate guidance documents covering patient involvement in:

- Pharmaceutical industry-led medicines R&D (5)
- Ethics committees
- Regulatory authorities (6)
- Health technology assessment (HTA) (7)

Each guidance suggests areas where at present there are opportunities for patient involvement. These guidances should be periodically reviewed and revised to reflect evolution.

The following values are recognized in the guidances and worked toward through the adoption of the suggested working practices. The values are:

Relevance: Patients have knowledge, perspectives and experiences that are unique and contribute to ethical deliberations.

Fairness: Patients have the same rights to contribute to the ethical review of clinical trials as other stakeholders and have access to knowledge and experiences that enable effective engagement.

Equity: Patient involvement in the ethical review process contributes to equity by seeking to understand the diverse needs of patients with particular health issues, balanced against the requirements of the industry.

Capacity building: Patient involvement processes address barriers to involving patients in ethical reviews and build capacity for patients and ethics committees to work together.

All subsequently developed guidances should be aligned with existing national legislation covering interactions as stated in the four EUPATI guidance documents.

#### Disclaimer identical in all four EUPATI guidances

EUPATI has developed these guidances for all stakeholders aiming to interact with patients on medicines research and development (R&D) throughout the medicines R&D lifecycle.

These guidance documents are not intended to be prescriptive and will not give detailed step-by-step advice. These guidances should be used according to specific circumstances, national legislation, or the unique needs of each interaction. These guidances should be adapted for individual requirements using best professional judgment.

Where these guidances offer advice on legal issues, it is not offered as a definitive legal interpretation and is not a substitute for formal legal advice. If formal advice is required, involved stakeholders should consult their respective legal department if available, or seek legal advice from competent sources. EUPATI will in no event be responsible for any outcomes of any nature resulting from the use of these guidances.

The EUPATI project received support from the Innovative Medicines Initiative Joint Undertaking under grant agreement n° 115334, resources of which are composed of financial contribution from the European Union's Seventh Framework Programme (FP7/2007-2013) and EFPIA companies.

### Specific guidance for patient involvement in ethical review of clinical trials

#### Introduction

To ensure optimal benefit for patients from a new medicine, and resulting commercial success, pharma companies focus the selection of compounds to develop and the definition of relevant research outcomes around the needs of patients with the respective disease. “Patient centricity” is a rapidly evolving and increasingly important element of pharma companies' business models. It requires new strategies, new organizational structures, and culture change across the pharma sector. It requires partnership with patient experts who are capable of providing advice on the value of treatments and on what health outcomes are relevant to patients. However, the concept of patient centricity is also relevant for other stakeholders in the medicines development process, especially for research ethics committees who advocate for the protection of patients in clinical trials.

Good clinical trial design is both ethical and scientifically sound. Design decisions include whether the new medicine is to be compared to another medicine or a placebo, how study participants should be selected, and what kind of tests and assessments are to be made (and how often). The risk of potentially harmful side effects needs to be balanced against the potential benefits for the patients taking part, such as early access to a new medicine, more intense diagnostics and supervision, and the chance to contribute to the development of new treatments for other patients with the same disease. Patients' judgements about such risks and benefits might be different to that of researchers: for instance, depending on the severity of the disease in question, they might be prepared to take a higher risk concerning potential side effects. In today's practice, the involvement of patients in these decisions is not standard—neither in clinical trials initiated by pharmaceutical or biotechnology companies nor in those initiated by academic institutions.

Clinical trials are subject to a framework of very strict laws. Before a clinical trial can start it needs approval from the competent authority which must ensure that all legal conditions are fulfilled, that the trial is scientifically sound, that the study medication is of proven quality and safe based on preclinical and—if available—previous clinical evidence; and that there is a favorable balance between expected benefits and risks. In parallel to the review by the national competent authority, one or more multi-disciplinary (research) ethics committees review the study protocol and related documents in order to safeguard the study participants. They ensure that the information to patients is comprehensive and understandable. They assess the balance between benefits and risks, ensure that this balance is acceptable, and that the trial is scientifically relevant for patients with the disease in question.

In most European countries patients, carers, or patient representatives are only marginally or not at all involved in the ethical and scientific review of clinical trials. In the national legislation of most European countries as well as in the new EU Clinical Trial Regulation (Regulation (EU) 536/2014) the involvement of patients in the definition of the ethical conditions for clinical trials and in the review provided by ethics committees is not clearly defined. The Regulation's Recital 18 states: “When determining the appropriate body or bodies (i.e., ethics committees), involved in application assessments, Member States should ensure the involvement of laypersons, in particular patients or patients' organisations” (8).

While patient involvement in R&D is a more and more accepted concept in the pharmaceutical and biotechnology industry, patient involvement in ethics committees is much disputed. Ethics committees are expert advisory groups providing advice on the ethical acceptability of research projects carried out in human beings. They have an obligation to the public to protect the research participants. To fulfill these obligations, ethics committee members need to be independent, neutral, objective and competent in scientific, ethical and methodological topics. The inclusion of a lay member is supposed to support this neutrality and to enlarge the scope of advice. Adding patient members to an ethics committee means a paradigm shift: the patient who represents those who will ultimately benefit from the research sits at the table, may—as a concerned party—overestimate the benefit or underestimate the risks in trial participation. However, the considerations underlying the concept of “patient centricity” in R&D are likely to also apply here: the outcome can be improved if the concerned party can provide their expert input. There is a need for a generally accepted guidance outlining the conditions for collaboration of ethics committees and patients in ethical review.

#### Scope

This guidance has been developed by the European Patient Academy on Therapeutic Innovation (EUPATI) for all stakeholders in medicines development involved in the ethical review of clinical research projects, with special emphasis on members of research ethics committees and patients/carers or patient representatives providing patient input.

**Figure 1:**
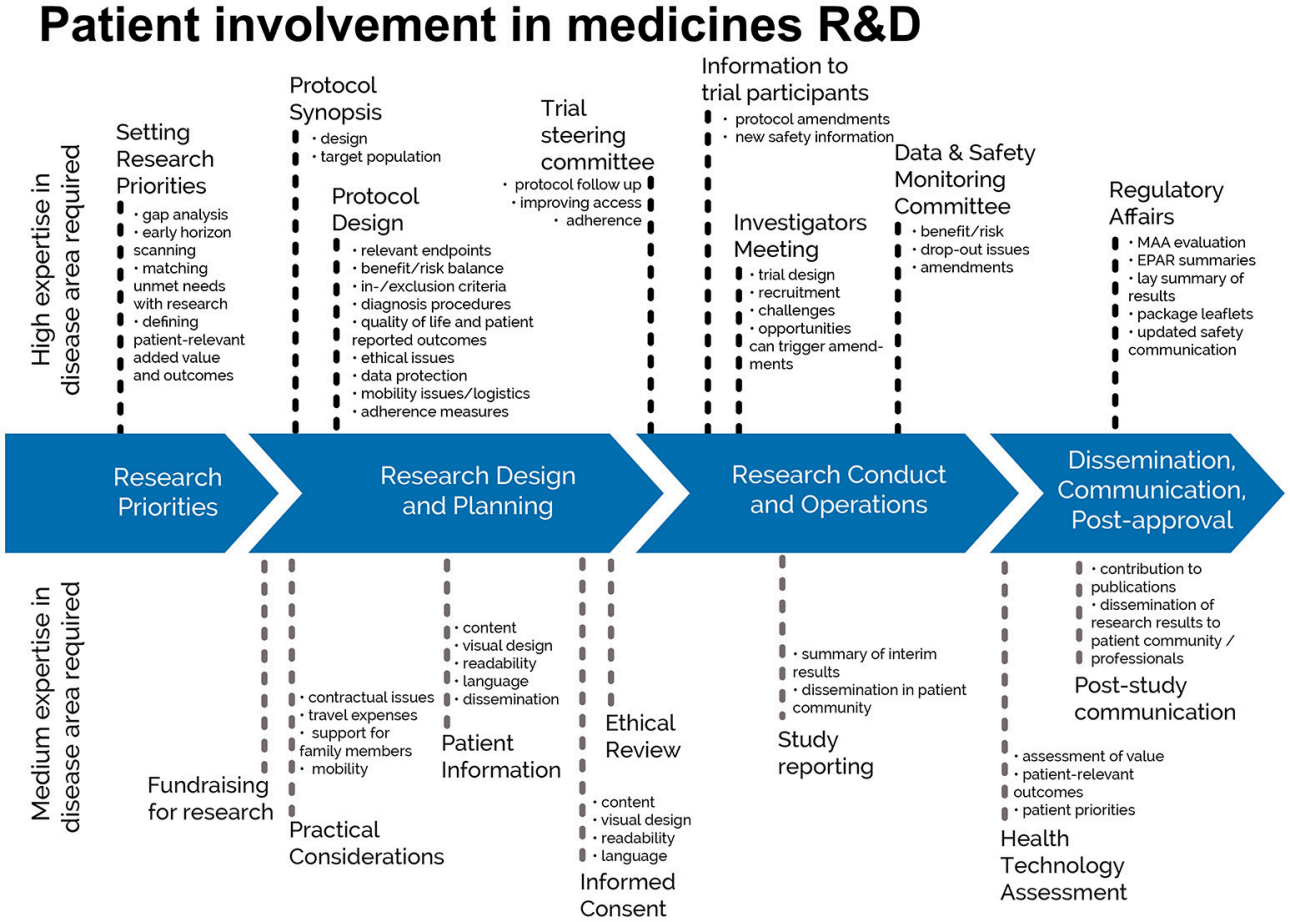
Patient involvement in medicines R&D: Patients can meaningfully contribute across the process of medicines R&D. This diagram distinguishes between areas in the R&D process that require a high level or a medium level of expertise in the respective indication. © EUPATI, under a Creative Commons licence CC BY-NC-SA 4.0. Used with permission.

**Figure 2:**
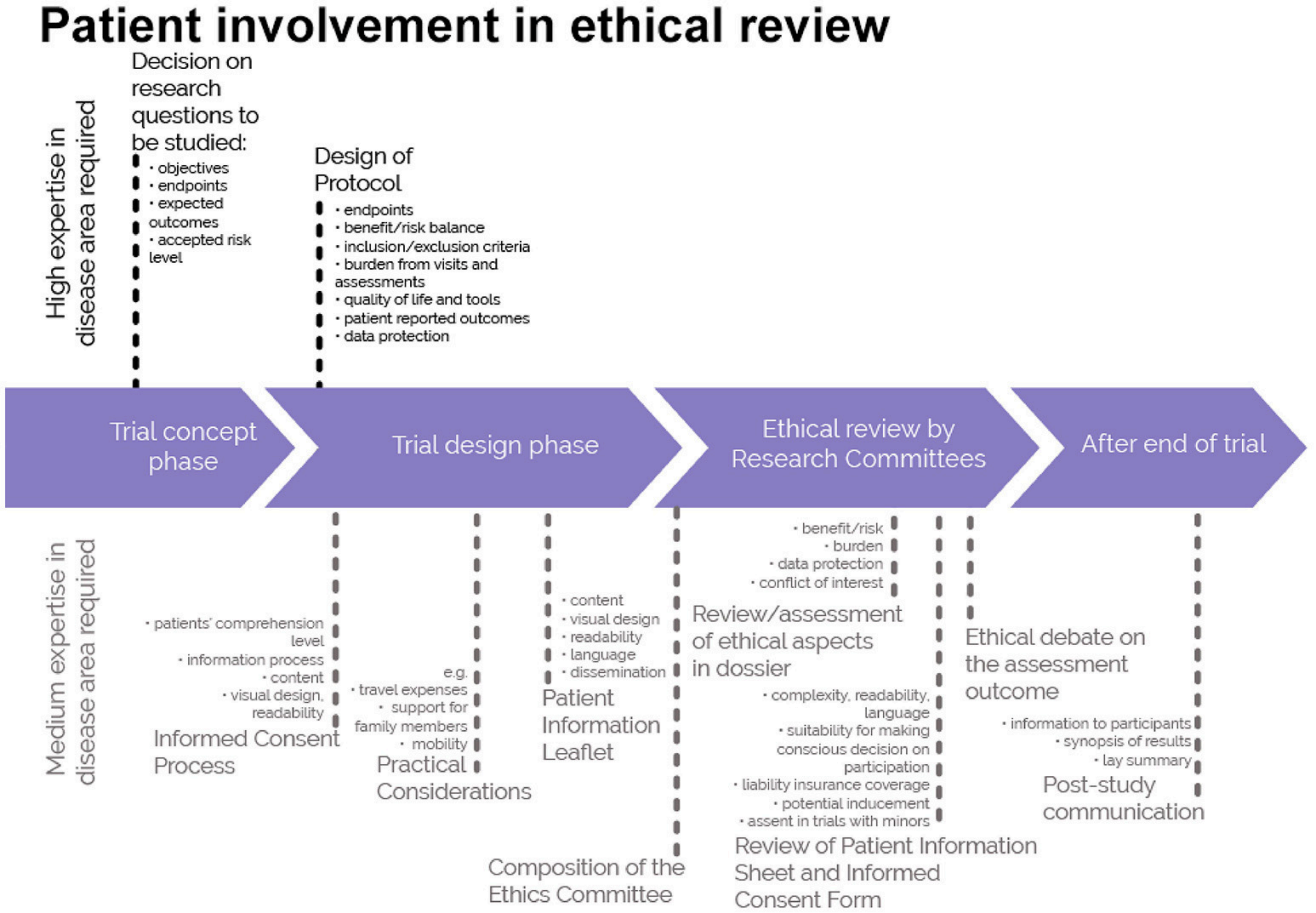
A roadmap where patient involvement may occur in ethical review of clinical trials. Patients can meaningfully contribute across the clinical trial process. This diagram distinguishes between areas in the clinical trial process that require a high level or a medium level of expertise in the respective indication. ©EUPATI, under a Creative Commons licence CC BY-NC-SA 4.0. Used with permission.

This guidance covers patient involvement in ethical review of clinical trials. Ethical aspects need to be considered in any step of the clinical trial—from definition of the research questions and protocol conditions, to informed consent preparation, to ethical review by ethics committees and to provision of information on trial results to the public. See Figures 1, 2. This guidance covers patient involvement in any of these steps, although special emphasis is given to patient involvement in research ethics committees.

This guidance is based on the discussions and conclusions from a multi-stakeholder roundtable discussion and a webinar on patient involvement in ethical review organized by EUPATI, contributions from national ethics committees, consultation within the EUPATI consortium and a comprehensive external consultation process.

#### Defining “patient”

The term “patient” is often used as a general, imprecise term that does not reflect the different types of input and experience required from patients, patient advocates and patient organizations in different collaborative processes.

In order to clarify terminology for potential roles of patient interaction presented in this and the other EUPATI guidance documents, we use the term “patient” which covers the following definitions:

- “Individual Patients” are persons with personal experience of living with a disease. They may or may not have technical knowledge in R&D or regulatory processes, but their main role is to contribute with their subjective disease and treatment experience.
- “Carers” are persons supporting individual patients such as family members as well as paid or volunteer helpers.
- “Patient Advocates” are persons who have the insight and experience in supporting a larger population of patients living with a specific disease. They may or may not be affiliated with an organization.
- “Patient Organisation Representatives” are persons who are mandated to represent and express the collective views of a patient organization on a specific issue or disease area.
- “Patient Experts,” in addition to disease-specific expertise, have the technical knowledge in R&D and/or regulatory affairs through training or experience, for example EUPATI Fellows who have been trained by EUPATI on the full spectrum of medicines R&D.

There may be reservations about involving individual patients in collaborative activities with stakeholders on grounds that their input will be subjective and open to criticism. However, EUPATI, in line with regulatory authorities, instills the value of equity by not excluding the involvement of individuals. It should be left to the discretion of the organization/s initiating the interaction to choose the most adequate patient representation in terms of which type of patient for which activity. Where an individual patient will be engaged it is suggested that the relevant patient organization, where one exists, be informed and/or consulted to provide support and/or advice.

The type of input and mandate of the involved person should be agreed in any collaborative process prior to engagement.

#### Current status of patient involvement in ethical review

Best practice examples have shown that patient involvement in ethical considerations concerning clinical trials as early as in the trial design and protocol preparation stage can be beneficial to strengthen the awareness about ethical issues in the research project. Involvement at this stage can ensure that the focus on the patient is maximized and the outcomes to be measured are relevant to patients. Guidance on this interaction is provided by the EUPATI “Guidance on patient involvement in industry-led medicines R&D” (5). Similarly, in clinical trials being driven by academia, patient experts could provide meaningful advice.

At the time of ethical review of the clinical trial by the ethics committee, the protocol details have been decided. Focus of this review is the acceptability of the specific benefit-risk balance, the patient protection elements and research site qualification as well as the information to patients during the informed consent process, by ethics committee members bringing in their respective expertise. The addition of patients' specific expertise can be a relevant expansion of a committee's expertise.

While participation of at least one lay person in ethics committees is longstanding practice and of undisputed value, the type and extent of patient involvement varies widely between—and even within—European Member States. In some countries patient representation is required by law and the conditions are clearly defined. In other countries individual ethics committees are just beginning to implement patient involvement within the frame of their statutes' flexibility on committee composition or because the law leaves it to the ethics committee to decide if they will involve a lay person or a patient representative. Different practices exist for the following reasons:

- Although there is appreciation of the benefit of patient involvement there is no agreement on the role and most suitable patient profile: patient expert, patient advocate, patient organization representative or individual patient.
- Finding patients willing to contribute to the ethical review is a challenge for ethics committees, and this is the case across Europe. There is no established match-making process.
- Involving patients with specific diseases can be logistically challenging, while involving patients who advise on all kinds of diseases requires a level of knowledge beyond their personal disease.
- There is disagreement about how far patients with a particular disease can and want to be representative for other patients with this disease, and whether there is potential for bias because of their personal interests. The independence of representatives from patient organizations has been questioned on the grounds that their personal interests and financial support from the pharmaceutical industry might lead to conflicts of interest.
- Pan-European capacity of suitable patient experts is currently scarce.

So far, a limited number of patient organizations decided to make efforts to identify and educate individual members for a role with relevant contributions in ethical review and specifically in an ethics committee.

As of 2020, the approval and performance of clinical trials will be governed by the European Clinical Trial Regulation 536/2014. Involvement of patients in the ethical review process is not stipulated in this Regulation, although the legislation states that lay persons, in particular patients or patient organizations, should be involved in the assessment of the clinical trial authorization application. The assessment process and the make-up of the assessing bodies (national competent authorities and ethics committees) are subject to national legislation, consequently the involvement of patients in the ethical review process will continue to vary from country to country.

#### Timing and nature of patient involvement in ethical review

Patients can be involved in the ethical review of clinical trials at different time points:

- Trial Concept Phase (handled by commercial or academic sponsor)
- Trial Design Phase (handled by commercial or academic sponsor)
- Ethical Review Phase [handled by ethics committee(s)]
- After End of Trial (handled by commercial or academic sponsor)

In the Trial Concept Phase patient experts can advise on ethical aspects of the trial such as:

- Assessment of preclinical data and/or background evidence
- Research questions, e.g., for specific indications, patient populations, etc.
- Defining the objectives of the trial to ensure its relevance for patients
- Inclusion and exclusion criteria of trial participants
- Acceptable/relevant endpoints
- The suitability of measurements and assessments, e.g., quality of life questionnaires and Patient Reported Outcomes
- Comparators (placebo or active comparator) and their acceptability for participants
- Acceptable risk levels: patients might have a specific opinion on the level of risk they are prepared to accept

*We recommend that patient experts should be involved in the Trial Concept Phase - whether a trial is being run by a company or academic centre - to optimise the scientific value of the trial and its viability*.

In the Trial Design Phase patient experts can advise on the specifics of the clinical trial that need to be defined in such a way that:

- A suitable number of participants can be recruited in an acceptable time frame
- The benefits of trial participation outweigh the risks
- The burden to participants is acceptable
- The care provided to participants is adequate
- Administration of the trial medication is as easy and reliable as possible
- Measurements and assessments are practical, acceptable to participants and reliable
- Patients will be informed of the trial results, even if stopped early
- The communities where the trial is performed will benefit from its results

Although patients can provide valuable input in many other aspects, a typical area of patient involvement in this phase is the development of the informed consent process including the preparation of the patient information sheet and informed consent form. Input from the kind of patient that these documents are developed for can improve their readability, user-friendliness, and completeness.

*We recommend that patient experts should be involved in the Trial Design Phase - whether a trial is being sponsored by a company or academic centre - to support the acceptability of the trial conditions for participants and the relevance of its outcome for the respective patient community*.

In the Ethical Review Phase, performed by one or more ethics committees, patient experts, or patient advocates can provide important input into the elements described above. In addition, patients can advise on local conditions for the trial such as:

- Assessment of the benefit/risk balance
- Fairness of inclusion and exclusion criteria
- Suitability of patient liability coverage (insurance)
- Data protection measures
- Potential conflicts of interest
- Readability and acceptability of the informed consent documentation
- Avoidance of inducement, for example ensuring that patient fees or travel expenses are appropriate
- How patient organizations can contribute to the patient information and recruitment processes

*We recommend that patient experts, patient organisation representatives or patient advocates who are knowledgeable about living with the disease in question should be involved in the review of clinical trials provided by ethics committees, to support trial participants' optimal protection*.

Sponsors sometimes involve patients in communication with trial participants after the end of the trial, but this has been very limited in the past. Under the new Clinical Trial Regulation, however, the results of every clinical trial will have to be communicated in a lay summary, to ensure transparency and to recognize the patient community's contribution to the trial. Patient input to lay summaries will be essential to ensure they are suitable and readable for patients.

*We recommend that commercial/academic sponsors involve patient experts or patient organisation representatives, knowledgeable about living with the disease in question, in the development of lay summaries to ensure they are non-biased, suitable and readable for patients*.

#### Practical aspects of patient involvement in ethics committees

National legislation outlines the constitution, organization and responsibilities of ethics committees, and reflects the roles of different types of ethics committees in the protection of trial participants and research integrity.

Different roles for patients in ethics committees can be considered:

- Full member of an ethics committee with equal rights and obligations as all other members
- External peer reviewer giving advice to the ethics committee members before their review meeting

The specific process for selection of the members of an ethics committee varies between countries and are defined by national legislation, responsible professional bodies or the ethics committee's own standard operating procedures.

#### Patients' level of expertise

Ethics committees should make a reasoned decision on the level of expertise they expect from their patient member(s):

- “Individual patients” with the disease in question, parents or carers of those patients, can provide valuable input to the patient information sheet and informed consent/assent form with a view from outside and can comment on aspects of a trial that will affect quality of life and the burden for participants. However, after some months of experience they might not be research-naïve anymore and it is argued that this could affect the value of their input. It can be difficult for research-naive patients to take part in discussion of other ethical topics that involve scientific and/or methodological complexity. The contributions of research-naïve patients without experience of the disease in question could be seen as comparable to those of lay persons.
- “Patient advocates” have an in-depth knowledge of living with the disease from their own experience and might have a level of understanding of research and medicines development for this disease. With each ethical review project they gain additional experience. The representativeness of their advice, however, might be limited by lack of in depth knowledge about cases beyond their own and perhaps a few other cases. Their contribution to ethical review of trials for other diseases will be limited to a general patient perspective.
- “Patient organization representatives” are either patients with the disease in question and/or actively engaged in a relevant patient organization and are exposed to the disease experience of many individuals. They are knowledgeable about the needs, desires and opinions of this community and thus will be relatively representative. Since patient organizations exist to support their members and to lobby for their interests it is important to ensure that the patient organization representative in the ethics committee is aware of his/her obligation to provide un-biased advice. Their contribution to ethical review of trials for other diseases will be limited to a general patient organization perspective.
- “Patient experts” (e.g., EUPATI Fellows) have personal experience of living with the disease and/or the combined knowledge from working with members of their patient organization. In addition, they have a comprehensive understanding of all aspects of the medicines development process, and can actively participate in all aspects of the ethical debate on the same level as the other ethics committee members. They are not joining the ethics committee in a representative role but have much exposure to other cases due to their activities in their patient organization. Their contribution to ethical review of trials for other diseases could also be valuable because of their knowledge of R&D.

*We recommend that patient experts, patient advocates or patient organisation representatives knowledgeable about living with the disease in question should be involved in the work of research ethics committees, preferably as full members, to extend their input beyond development of the patient information sheet and informed consent form*.

#### Finding supportive patients and interested ethics committees

Ethics committees report that it is difficult to find patients willing to participate, and in particular to find patients with the expected level of expertise. Involvement of a “generic” patient representative reviewing trials for all kinds of diseases makes finding patient members easier but this has disadvantages as described above. Identifying patient members for specific diseases and bringing them to ethics committee meetings can be a logistical challenge. However, patients can participate in ethics committee meetings via tele- or web-conference. Alternatively, patients can be asked to provide their written comments before the ethics committee meeting but this means that the impact of patients on the ethical debate during the meeting is missed.

There are a number of options for ethics committees to identify interested patients and for interested patients to join an ethics committee:

- Ethics committees can establish collaboration and enable ethical review education opportunities with (umbrella-) patient organizations
- Advertisement
- Use of existing contacts
- Unsolicited applications from patients
- Supporting the development of a national match-making platform jointly with academic and commercial sponsors to facilitate collaboration with interested patients with different diseases and different levels of expertise.

*We recommend that individual ethics committees develop a database of patients willing to join the ethical review process and we encourage ethics committees to join forces to establish a joint database, e.g., on national or regional level*.

*We recommend that patient organisations create a database of members interested and educated in ethical review of clinical trials. Patient organisations should communicate the existence of this database to the national ethics committees*.

#### Conditions for patient involvement in ethics committees

The conditions for patient involvement in the work of an ethics committee should be communicated to interested patients or patient representatives to ensure smooth and efficient collaboration.

#### Written agreement

A written agreement should be signed by both parties containing a clear description of the role of the patient in the ethical review process. The agreement should specify the legal and regulatory conditions, working procedures, ground rules, and conflict resolution procedures, frequencies of interaction, mutual obligations including confidentiality, liability (insurance) protection, resource requirements, and timelines as well as the mechanism for payment/reimbursement of expenses and any other benefits.

*To ensure clarity about the collaboration between ethics committees and participating patients we recommend signing a written agreement before the start of the collaboration*.

#### Transparency

As with all members of an ethics committee, patient members in ethics committees should ensure they are transparent about their own (and/or their patient organization's) professional interests and financial support.

*We recommend that patient members should sign the same Declaration of Interest as the other ethics committee members, to list potential conflicts of interest such as professional involvement and financial interests in other organisations and personal and professional (if the patient is a patient organisation representative) funding sources*.

#### Representativeness

Representativeness of the patient members' advice is an important aspect for both the ethics committee and the patient community they are representing. Only a limited number of patient organizations have systematically compiled information relevant for the ethical review of a clinical trial in their area of indication and decided on a member interested and suitable to represent the organization in an ethics committee.

*We recommend that patient organisations identify members interested in representing the organisation in an ethics committee and ensure that these members receive comprehensive information about the community's treatment needs, quality of life deficiencies, and day-to-day life conditions*.

*We recommend that patient organisations implement a mechanism to exchange experiences which their members develop in ethics committees while respecting the patient members' confidentiality obligations*.

#### Appointment, introduction, and training

The appointment process and introduction of patient members should follow the standard rules of the respective ethics committee.

Participating in the ethical review in an ethics committee is for many patients and patient organization representatives a new experience. Debating with experts in their field might be intimidating and can lead to a lack of contributions: it is important that the mere presence of patient representation is not seen as a given endorsement to committee decisions. To support real engagement, the capacity of patients experienced in providing advice to ethics committees needs to be systematically increased. This should include a comprehensive introduction into the work of an ethics committee member and continuous professional development initiatives, even if his/her involvement is limited to contributions relevant to their disease area.

*We recommend that patient members receive a comprehensive introduction and appropriate continuous training independent of the frequency of their participation in ethical review*.

#### Compensation

It should be recognized that in many situations patients involved in activities do so voluntarily either as an individual but also when a member of an organization. Consideration should therefore be given to:

- Compensate for their total time invested plus expenses
- Any compensation offered should be fair and appropriate for the type of engagement. Ideally travel costs would be paid directly by the organizing partner, rather than being reimbursed
- Covering the costs incurred by patient organizations when identifying or supporting patients for involvement in activities (i.e., peer support groups, training, and preparation) should also be considered
- Help organize the logistics of patient participation, including travel and/or accommodation.

Compensation also includes indirect benefits in kind (such as a patient organization providing services free of charge) or any other non-financial benefits in kind provided to the patient/patient organization (such as training sessions, the setting up of web sites).

All parties should be transparent about any compensation arrangements.

## End of guidance text

## Discussion

In the European Union ethical review of a clinical research project and decision on its ethical acceptance is the responsibility of ethics committees which are established and functioning according to national legislation. The Clinical Trials Directive (3) and the new Clinical Trials Regulation (4) only define the required outcome of the national ethical review system and the maximum time frames for the review process, but not the constitution of the ethics committees and their procedures. Thus, all EU Member States and all countries outside the EU define their ethics committee systems independently. While the involvement of a lay member in the review process became an accepted principle in the 1990s as part of the development of Good Clinical Practice Guidelines, the concept of involving patients gained attention only recently and few countries have established rules on such participation. But even in countries where patient representation in ethics committees is regulated by law, different conclusions were drawn concerning the usefulness of such involvement. As has been pointed out in the EUPATI round table and webinar on patient involvement in ethics committees by both, ethics committee members and patients who participate in ethics committees, patients' contribution is limited when there is lack of knowledge about the clinical trial methodology and its ethical implications in the medicines development process.

The fact finding and discussions held in EUPATI also revealed diverging opinions on whether patients should be members of ethics committees or not. Criticism is based on the arguments that patients are “concerned parties” and their presence would undermine the objectivity of the discussion and on the challenges of finding knowledgeable patients who understand the methodological problems, who can provide a representative opinion and feel comfortable in debating with scientific and ethical experts. However, there are countries where patient involvement in ethics committees works very well, where patients are providing valuable insight into living with the disease and the acceptability of proposed studies, and who are helping improve the clarity of information to study participants.

An important discussion during this guidance's development raised the question about the representativeness of patients in ethics committees: can individual patients talk on behalf of other patients with this disease? Do patient organization representatives—not being sick themselves—or “generic” patients provide enough genuine expertise on how it is to live with the disease? As best possible solution it was proposed that patient organizations systematically collect and document experiences made by their members that might be relevant for the design and organization of clinical trials for a specific condition and that this information be made available to all patients or patient representatives participating in an ethics committee.

The scope of the guidance was deliberately chosen to present guidance on a broader topic than involvement of patients in ethics committees: the involvement of patients in ethical considerations throughout the entire medicines development as presented in Figure 1 and specifically in the ethical review of the clinical trial process as described in Figure 2. The review by ethics committees occurs at the end of the protocol development process. Many important decisions, however, are made earlier and benefit from patients' contributions based on their unique experience. Commercial and academic sponsors are encouraged to enable patient input at these earlier development stages. The EUPATI definition of patient levels of expertise will further help to identify the most suitable patients for the different stages of medicines and protocol development.

## Conclusion

Involvement of patients in ethical review should occur throughout the entire R&D process, and in particular in the whole clinical trial process from protocol development to lay summary writing, including project review by ethics committees. Based on this Guidance's definition of patient categories and their recommended involvement in the different development phases clinical researchers and ethics committees can now clearly define which type of patient expertise they are looking for. The provided guidance for all relevant practical aspects of patient involvement in ethics committees will help ethics committees to properly adapt their processes and governance infrastructure to integrate patients as valued members. Patient organizations are encouraged to work on preparedness of their members to provide relevant input and enable transparency on potential conflicts of interests in line with the obligations of all other members of an ethics committee. The Guidance presented here provides useful insight into what needs to be considered to facilitate patient involvement, as well as practical solutions for smooth and efficient collaboration. It can serve as the standard on which all further codes should be based.

## Author contributions

IK drafted the article and the guidance document with significant input from AH, WS, DH, MM, KW.

### Conflict of interest statement

The authors declare that the research was conducted in the absence of any commercial or financial relationships that could be construed as a potential conflict of interest.

## Footnotes

^1^For further information please visit: www.eupati.eu

^2^Consumers are recognised as stakeholders in the healthcare dialogue. The scope of EUPATI focuses on patients rather than consumers. This is reflected in the educational material and guidance documents.
